# Time course and regional heterogeneity of hamstring muscle thickness after maximal concentric exercise in men and women

**DOI:** 10.1007/s00421-026-06223-8

**Published:** 2026-04-13

**Authors:** Chrysostomos Sahinis, Konstantinos Pavlidis, Eleftherios Kellis

**Affiliations:** Laboratory of Neuromechanics, Department of Physical Education and Sport Sciences at Serres, Aristotle University of Thessaloniki, TEFAA, Serres, 62100 Greece

**Keywords:** Hamstrings, Ultrasound, Muscle swelling, Recovery, Sex

## Abstract

**Purpose:**

This study examined the early time course of hamstring thickness changes after acute maximal concentric knee-flexions across multiple measurement sites in men and women.

**Methods:**

Twenty-two young adults (11 men, 11 women) performed 50 maximal unilateral concentric knee-flexions at 120°·s⁻¹. Muscle thickness of the biceps femoris (BF) and semitendinosus (ST) was assessed using ultrasound at proximal, middle, and distal sites at baseline and repeatedly during 30-min of recovery.

**Results:**

Linear mixed-effects models showed that thickness increased rapidly, peaking immediately post-exercise or at 5-min (mean relative change: 10.4 ± 4.9%), and returned toward baseline by 15–30-min. Absolute thickness increases differed between muscles and sites (*p* < .05), with larger responses observed in BF than ST (*d* = 0.61–3.23) and the greatest change at the middle site (*d* = 1.00–4.04). In contrast, relative thickness changes were largely comparable across muscles, sites, and sexes (*p* > .05). However, considerable inter-individual variability was found in relative thickness changes among measurement sites with the distal regions most often contributed the largest share of total swelling (BF 45.5%, ST 54.5%).

**Conclusion:**

Maximal concentric exercise induces transient hamstring thickness changes. Although relative thickness responses were similar across sexes, muscles, and regions, substantial inter-individual variability in regional patterns suggests that the hamstrings may not respond as a uniform unit acutely following exercise.

**Supplementary Information:**

The online version contains supplementary material available at 10.1007/s00421-026-06223-8.

## Introduction

Muscle swelling refers to the transient increase in muscle thickness observed after exercise (Ploutz-Snyder et al. 1995; Csapo et al. 2011; Vieira et al. 2018). It is mainly attributed to rapid fluid shifts and hemodynamic changes during and after repeated contractions, including an increase in intramuscular blood volume (Leyk et al. 1994) driven by metabolite accumulation and the resulting osmotic gradients (Lanne et al. 1959; Ploutz-Snyder et al. 1995; Finan and Guilak 2010). Acute increases in muscle thickness have been used to infer which muscles (or regions within a muscle) were more strongly engaged by a given exercise, under the assumption that greater local demand is accompanied by greater local hyperemia and fluid accumulation (Vieira et al. 2018; Kassiano et al. 2023; Arima et al. 2023). Some studies report associations between acute post-exercise size changes and longer-term hypertrophic adaptations, suggesting that early responses may contain information relevant to training adaptation without necessarily implying causation (Wakahara et al. 2012; Hirono et al. 2022).

Immediately after exercise, muscle size increase rapidly (Ploutz-Snyder et al. 1995; Csapo et al. 2011; Freitas et al. 2020; Arima et al. 2023), followed by a partial resolution during early recovery as perfusion, fluid distribution, and intramuscular pressure change over time (Ploutz-Snyder et al. 1995; Csapo et al. 2011). However, many studies have assessed swelling at only a single post-exercise time point (Vieira et al. 2018; Kassiano et al. 2023), limiting inference about the time course of recovery and complicating comparisons across protocols and muscle groups.

Acute responses to resistance exercise are unlikely to be uniform along the muscle length (Sahinis et al. 2025c). Muscle activation, fascicle behavior, and local strain distributions can vary regionally during resistance exercise, producing non-uniform metabolic stress within a single muscle belly (Vieira et al. 2018; Sahinis et al. 2025c; Trinchi et al. 2025). For example, Vieira et al. (2018) found greater increase in muscle thickness in the middle than distal site of vastus lateralis after exercise. This may be particularly relevant in the hamstrings, which display intra- and inter-muscular heterogeneity in architectural features, such as fascicle length, pennation angle, and tendon/aponeurosis dimensions (Kellis et al. 2010; Kellis 2018) together with differences in muscle activation (Sahinis et al. 2024, 2025b). Overall, these factors may influence strain distribution and metabolic stress during exercise, potentially leading to distinct acute thickness responses within and between the muscles.

Women remain underrepresented in many resistance-exercise physiology studies (Roberts et al. 2020; Refalo et al. 2025). Sex-related differences in body size, muscle morphology, testosterone levels and vascular responses (Liu et al. 2010; Vingren et al. 2010; Green et al. 2016; Behan et al. 2018) could plausibly influence the magnitude or time course of post-exercise swelling. For example, greater muscle size in males may permit larger absolute increases in thickness associated with exercise-induced fluid shifts, whereas sex-related differences in vascular reactivity and perfusion could influence the rate of intramuscular fluid accumulation and clearance during recovery. Previous reviews have found no differences in relative muscle size adaptations following resistance training however such effects are not established acutely (Abe et al. 2000; Roberts et al. 2020; Refalo et al. 2025).

In the hamstrings, research on individual activation patterns has mainly relied on EMG amplitude providing insight into between-muscle activation patterns (Sahinis et al. 2025a; Morin et al. 2025). In contrast, far less is known about the acute thickness response of the hamstrings after exercise, particularly its early time course and whether this response varies between synergistic muscles or measurement sites. Therefore, the present study quantified the early time course of exercise-induced hamstring swelling measured pre-exercise and repeatedly during the first 30-min of recovery in males and females. Muscle thickness was assessed using ultrasound in biceps femoris (BF) and semitendinosus (ST) at multiple sites (proximal, middle, distal) to capture potential regional heterogeneity. Identifying regional and sex-specific swelling patterns will allow practitioner and coaches to develop site-specific rehabilitation protocols for the hamstrings. Based on previous evidence (Csapo et al. 2011; Vieira et al. 2018; Kellis 2018; Refalo et al. 2025), it was hypothesized that: (1) hamstring thickness would peak within 5-min post-exercise and return to baseline by 30-min; (2) absolute swelling would exhibit regional heterogeneity, and would vary between the hamstrings muscles; and (3) while men would show larger absolute increases in muscle size, the relative response after exercise would be similar between sexes.

## Materials and methods

### Participants

An a priori power analysis was performed in G*Power (v3.1) for an F-test using ANOVA: repeated measures, within–between interaction (two groups). The analysis assumed α = 0.05, power (1 − β) = 0.95, correlation among repeated measures *r* = .50, and nonsphericity correction ε = 0.60. The effect size was set to f = 0.29, based on a previous study (Vieira et al. 2018). Under these assumptions, the required total sample size was 10 participants. Twenty-two young adults (11 men and 11 women) took part in the study (mean ± SD, men: age 22.3 ± 1.8 y, mass 84.5 ± 6.7 kg, height 1.81 ± 0.09 m; women: age 21.8 ± 1.3 y, mass 70.3 ± 5.3 kg, height 1.69 ± 0.07 m). All participants provided written informed consent before testing. Eligibility required no lower-limb injury in the previous 12 months, including any hamstring strain or knee-related muscle/ligament injury. Participants were asked to avoid vigorous physical activity for 24-h before the laboratory visit. Ethical approval was granted by the University Institutional Review Board (ERC-006/2022).

### Experimental procedures

**Fig. 1:**
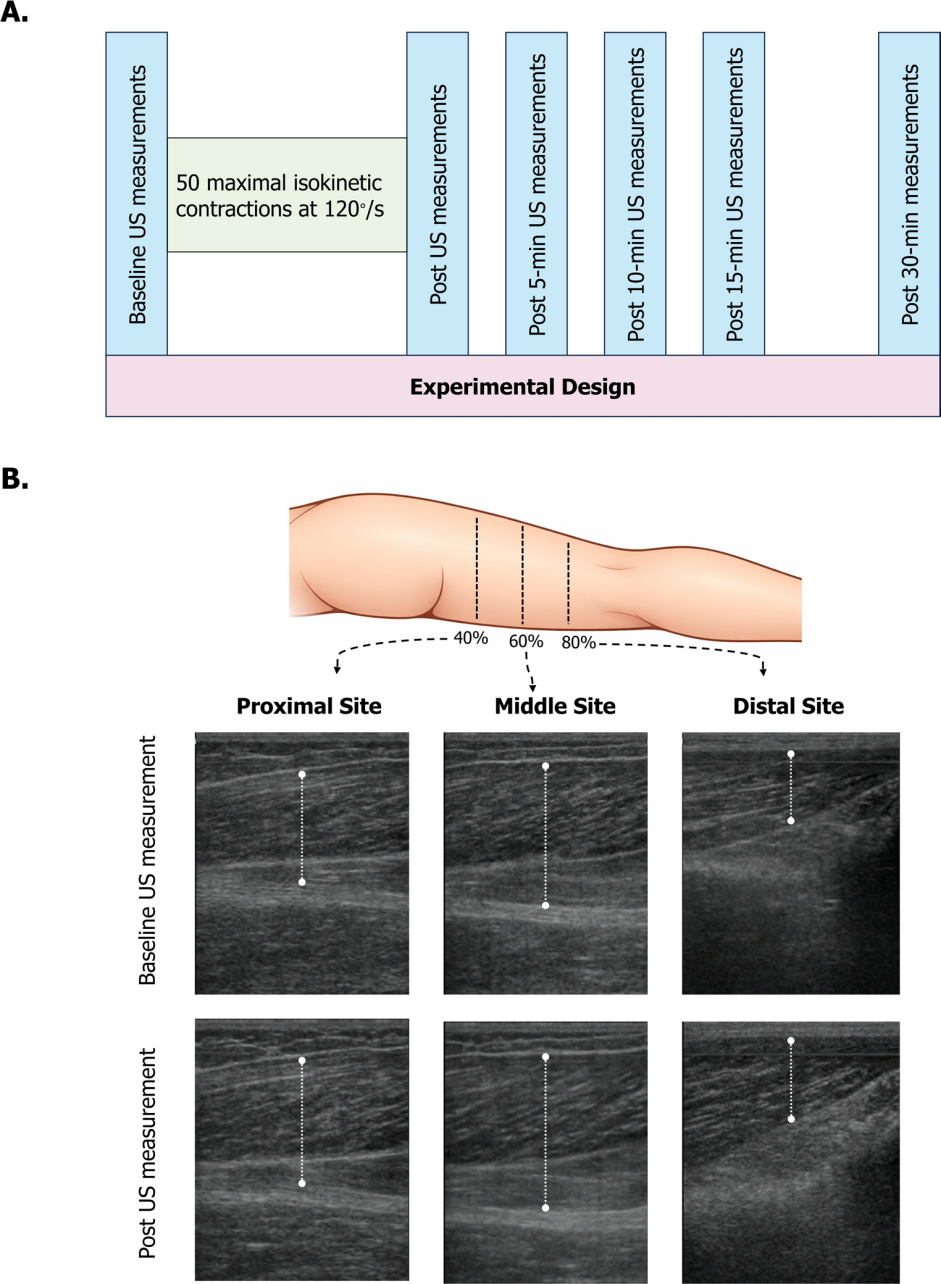
**A** Experimental design and representative ultrasound images. Protocol timeline: baseline ultrasound (US) measurements were collected, followed by 50 maximal isokinetic contractions at 120°/s, and repeated US measurements immediately post-exercise and at 5-, 10-, 15-, and 30-min post-exercise. **B** Schematic representation of the US measurement sites along the femur length. Representative B-mode US images of biceps femoris acquired at the proximal (40%), middle (60%), and distal site (80%) at baseline (top row) and immediately post-exercise (bottom row)

The study used a single-session repeated-measures design to quantify acute changes in muscle thickness of the long head of BF and ST along the muscle length. Participants performed 50 maximal unilateral concentric knee-flexion contractions with the right leg on an isokinetic dynamometer at 120°·s⁻¹ similar to previous work (Vieira et al. 2018). A concentric-only protocol was selected to characterize the early post-exercise thickness response under controlled isokinetic conditions while minimizing the delayed damage-related swelling more commonly associated with eccentric exercise. Ultrasound measurements were collected at baseline, immediately post-exercise, and at 5-, 10-, 15-, and 30-min post-exercise (Fig. 1A).

### Ultrasound assessment

Muscle thickness was assessed using B-mode ultrasound because it enables rapid, noninvasive imaging that is feasible for repeated measurements within short recovery intervals (Kellis et al. 2009; Sahinis and Kellis 2024a). Although magnetic resonance imaging can quantify muscle volume, it is less practical for repeated within-session assessments due to longer acquisition time and limited accessibility. Hamstring ultrasound images were acquired with participants lying prone (hips neutral, knees extended) by two investigators (C.S. and K.P.) using two ultrasound systems (SSD-3500, ALOKA, Japan; and GE LOGIQ 400 CL PRO, GE Medical Systems, U.K.) with a 10-MHz linear-array transducer (6-cm footprint). Participants remained in this position for 10 min before baseline imaging to standardize posture and minimize transient fluid shifts. To minimize delays between muscles and time points, the two systems were used in parallel. For each participant, one investigator consistently imaged one muscle (BF or ST) across all time points, while the second investigator imaged the other muscle. The allocation of muscle to system/investigator was randomized across participants but remained fixed within participant. All imaging settings were standardized and kept constant across participants (depth 80 mm, gain 59 dB, dynamic range 5 dB) (Kellis and Sahinis 2022; Sahinis and Kellis 2024a).

Femur length of the right limb was first measured as the distance between the greater trochanter and the lateral femoral condyle using an anthropometric tape. Longitudinal and transverse scans were then used to identify the approximate trajectory of each hamstring muscle and the proximal and distal musculotendinous junctions (Kellis and Sahinis 2022). These landmarks were marked on the skin to facilitate consistent probe placement and plane reproduction across time points and participants. Muscle thickness was assessed at three locations corresponding to 40% (proximal site, close to hip joint), 60% (middle site), and 80% (distal site, close to knee joint) of femur length. At each site, an axial line was drawn perpendicular to the limb axis, the probe was then placed at the line’s midpoint, aligned with the muscle’s longitudinal axis, and held perpendicular to the skin. A generous layer of water-soluble transmission gel (Transsound, EF Medica Srl, ) was applied and only minimal pressure was used to avoid compressing the tissue while maintaining acoustic coupling. Participants were instructed to remain fully relaxed throughout image acquisition. Relaxation was verified in real time by confirming the absence of visible contraction (e.g., fascicle shortening or muscle bulging) on the ultrasound image. Immediately after capture, images were screened by two investigators (C.S. and K.P.) for (i) clear visualization of superficial and deep aponeuroses as continuous hyperechoic bands, (ii) absence of motion artefacts, and (iii) adequate contrast for border identification. Scans were repeated if these criteria were not met. The acquisition of ultrasound images was randomized across measurement sites. All images were analyzed by a single examiner (C.S.) blinded to participant identity and time point. Muscle thickness was quantified in ImageJ (NIH, USA, v1.53e) as the distance between the superficial and deep aponeuroses in the center of each ultrasound image (Fig. 1B). Intra-rater, inter-rater, and inter-device reliability were assessed and are reported in Supplementary Material 1.

### Isokinetic dynamometry

The exercise bout was performed on an isokinetic dynamometer (Humac Norm, CSMi, USA), which was calibrated before testing according to the manufacturer’s instructions. Participants were positioned prone on the dynamometer chair with straps secured across the pelvis to minimize extraneous movement. The lateral femoral epicondyle was aligned with the dynamometer axis of rotation, and the lever arm attachment was secured ~ 5 cm proximal to the lateral malleolus. Before the main protocol, participants completed a standardized warm-up consisting of five submaximal–to–moderate isokinetic knee-flexions at 120°·s⁻¹ over a 0–90° range of motion (0° = full knee extension). During the fatiguing exercise protocol, participants were instructed to perform each repetition with maximal effort, flexing the knee “as hard as possible” through the prescribed range of motion, and then to fully relax while gravity returned the limb to the starting position. A total of 50 maximal concentric consecutives repetitions were completed (Vieira et al. 2018). Standardized verbal encouragement was provided by the same investigator. Immediately after the final repetition, participants were removed from the dynamometer and post-exercise ultrasound imaging commenced, with repositioning to the ultrasound setup taking ~ 1-min. The full visit was completed in ~ 50–60 min.

Fatigue index was quantified as the percentage decrease in peak torque from the first 5 to the last 5 repetitions: percent decrease = [ 1 − (last 5 /first 5)] × 100 [1 − (last 5/ first 5)] × 100 (Pincivero et al. 2003). Total mechanical work performed during the exercise protocol was also calculated by computing the integral of torque over the angular displacement and expressed in Joules (J). Total work was obtained by summing the work performed across all contractions.

### Statistics

Statistical analyses were performed using R (version 4.3.0, Vienna, Austria). Sex differences in fatigue index and total mechanical work were evaluated using an independent samples t-test. Prior to analysis, the normality of values was assessed using Shapiro–Wilk tests. To determine whether the magnitude of swelling was associated with mechanical workload, Pearson product–moment correlation analyses were performed between total mechanical work and the percentage change in muscle thickness post exercise at each muscle and measurement site (Vieira et al. 2018). The strength of correlations was interpreted using conventional thresholds (|r| < 0.30 small, 0.30–0.50 moderate, > 0.50 large).

Two outcomes were analyzed: (i) absolute thickness (mm) and (ii) relative thickness change (%) (*Δ*Thikness = [(timepoint − Pre) / Pre] × 100). For the primary inferential analyses, linear mixed-effects models were fitted with fixed effects of *Sex* (male, female), *Muscle* (BF, ST), *Site* (proximal, middle, distal), and *Time* (pre-exercise, post-exercise, 5-, 10-, 15-, 30-min post-exercise) with *Participant* included as a random intercept. Fixed effects were evaluated using Type III Wald χ^2^ tests with sum-to-zero contrasts, and statistical significance was set at *p* < .05. Post-hoc comparisons were conducted using model-based estimated marginal means with Holm adjustment. Descriptive data are reported as mean ± SD with 95% confidence intervals, otherwise stated. Effect sizes were expressed as Cohen’s d and interpreted as 0.2 small, 0.5 medium and 0.8 large. Model assumptions were assessed by inspection of residual and Q–Q plots.

To characterize the distribution of muscle swelling, the site-specific change in muscle thickness (proximal, middle, distal) was expressed as a percentage of the participant’s total thickness change summed across sites (shares summing to 100%) only at post-exercise and 5-min post-exercise as most participants presented the maximum changes in muscle thickness. Because percentage normalization can exaggerate relative differences when the total change is small, we restricted this analysis to these peak-response time points. Participants were classified by the site presented the greatest contribution (largest percentage change) and ties was classified as co-dominant, although none occurred. Inter-individual variability was quantified in two ways. First, site-specific deviation was calculated for each participant as the absolute difference (percentage points, pp) between their regional share and the group median share for that site. Second, overall profile mismatch was summarized using the total variation distance (D), calculated as: D = 0.5 × (|p_i, prox − p̃_prox| + |p_i, mid − p̃_mid| + |p_i, dis − p̃_dis|), where p_i, site is the participant’s site-specific share and p̃_site is the group median share for that site. (D) represents the percentage of the swelling distribution that would need to be reallocated across sites for a participant to match the group median pattern. This approach was used to describe the within-participant distribution of absolute thickness change across sites rather than to compare swelling magnitude between regions.

Time-to-peak swelling was defined for each participant × muscle × site interaction as the time point (post-exercise, 5-, 10-, 15-, or 30-min) at which ΔThickness was maximal (the largest observed increase relative to baseline). Because later peaks (≥ 10-min) were sparse, time-to-peak was modeled as a three-level categorical outcome (Post, 5 min, ≥ 10 min) using a multinomial mixed-effects model with *Sex*, *Muscle*, and *Site* as fixed and *Participant* as a random intercept.

## Results

### Fatigue index and total mechanical work

Fatigue index did not differ (t_(20)_ = 1.37, *p* = .19) between sexes (males: 47.3 ± 5.0% and female: 44.6 ± 4.2%). However, total mechanical work produced during the exercise bout was greater (t_(20)_ = 4.13, *p* = .0005, d = 1.76) in males than females (mean difference = 965.15 J, 95% CI [477.63, 1452.67]).

### Absolute thickness

**Fig. 2:**
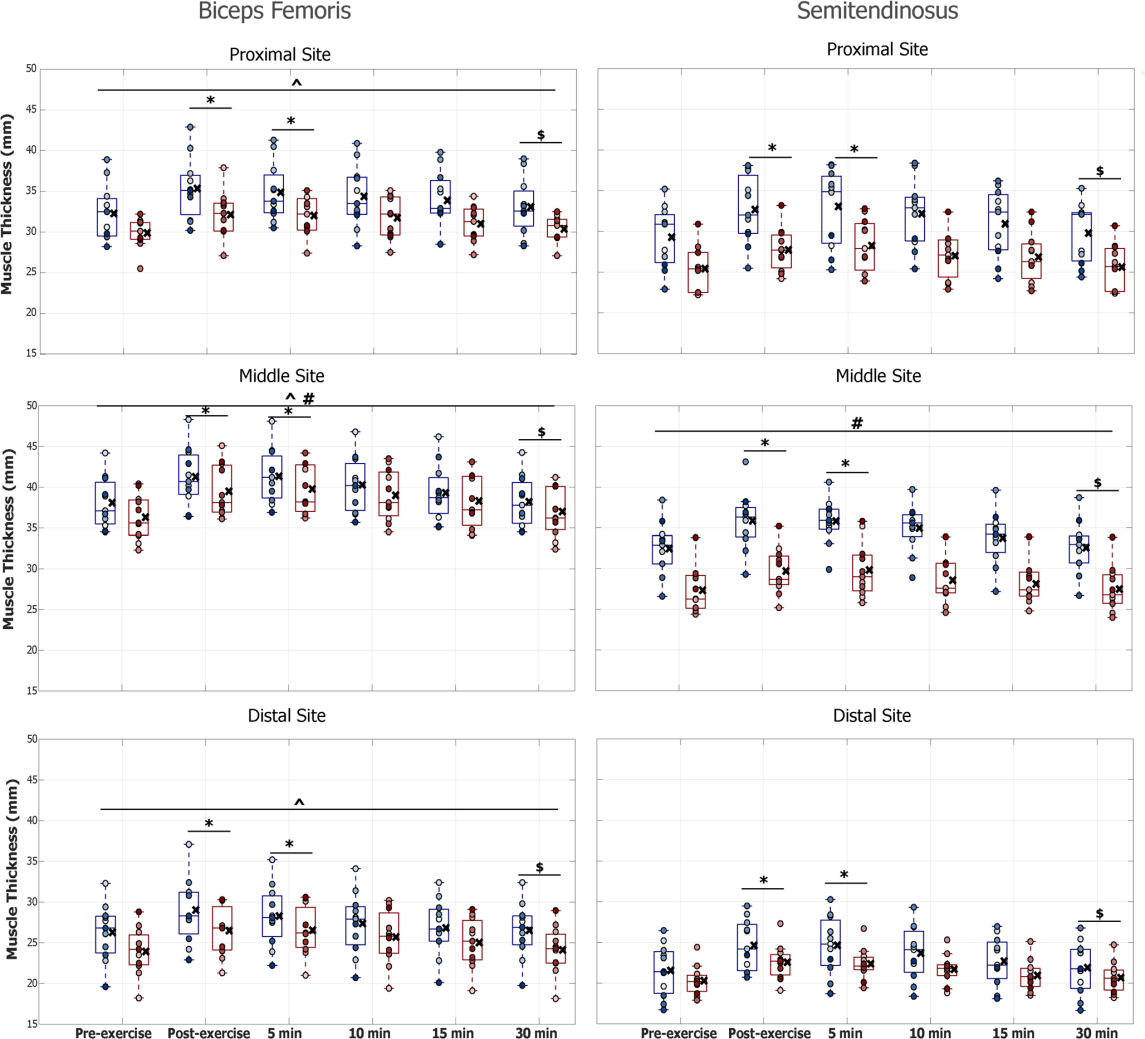
Absolute muscle thickness (mm) of biceps femoris (left column) and semitendinosus (right column) at the proximal, middle, and distal measurement sites pre-exercise, immediately post-exercise and at 5-, 10-, 15-, and 30-min post-exercise. Blue and red denote the two sex groups (men and women, respectively). Box boundaries represent the 25_th_ and 75_th_ percentiles (interquartile range), the horizontal line is the median, whiskers extend to values within 1.5 × IQR, circles show individual participants, and × denotes the mean. Individual participant points are color-coded. * Significant different than pre-exercise (*p* < .05); $ significant different than post-exercise (*p* < .05); ^ significant different than ST (*p* < .05); # significant different than proximal and distal sites (*p* < .05)

Analysis revealed significant main effect for *Time* (χ²_(5)_ = 103.13, *p* < .001), *Muscle*, (χ²_(1)_ = 537.31, *p* < .001), *Site* (χ²_(2)_ = 1746.42, *p* < .001), and *Sex* (χ²_(1)_ = 15.21, *p* < .001) for absolute muscle thickness. In addition, significant *Sex* × *Muscle* (χ²_(1)_ = 15.43, *p* < .001), *Sex* × *Site*, (χ²_(2)_ = 10.87, *p* = .004), *Muscle* × *Site*, (χ²_(2)_ = 88.73, *p* < .001), and *Sex* × *Muscle* × *Site* interaction were also found (χ²_(2)_ = 12.91, *p* = .002) (Fig. 2). Collapsed across *Sex*, *Muscle*, and *Site*, muscle thickness increased from pre-exercise (28.5 ± 6.3 mm, 95% CI [27.4, 29.6]) to post-exercise (31.3 ± 6.7 mm, 95% CI [30.2, 32.5]) and remained increased at 5-min (31.3 ± 6.7 mm, 95% CI [30.2, 32.4]) before approaching baseline by 30-min (28.8 ± 6.3, 95% CI [27.7, 29.9]). Post-hoc tests showed that muscle thickness at immediately post-exercise and 5-min post-exercise was greater than pre-exercise across muscles, sites, and both sexes (*p* ≤ .047, *d* = 1.48–4.46). In contrast, muscle thickness at 15- and 30-min post-exercise did not differ from pre-exercise (*p* ≥ .05). Also, muscle thickness at 30-min was lower than post-exercise values (*p* ≤ .037, *d* = − 3.09 to − 1.68). Across conditions, females had lower muscle thickness than males (*p* < .001, *d* = − 1.68 to − 0.34). BF was thicker than ST (*p* < .001, *d* = − 3.23 to − 0.61), and muscle thickness differed across sites, with the middle site greater than proximal and distal (middle > proximal > distal; *p* < .001, *d* = 1.00–4.04). Full descriptive statistics can be found on Supplementary File 2.

### Relative change (Δ thickness)

**Fig. 3:**
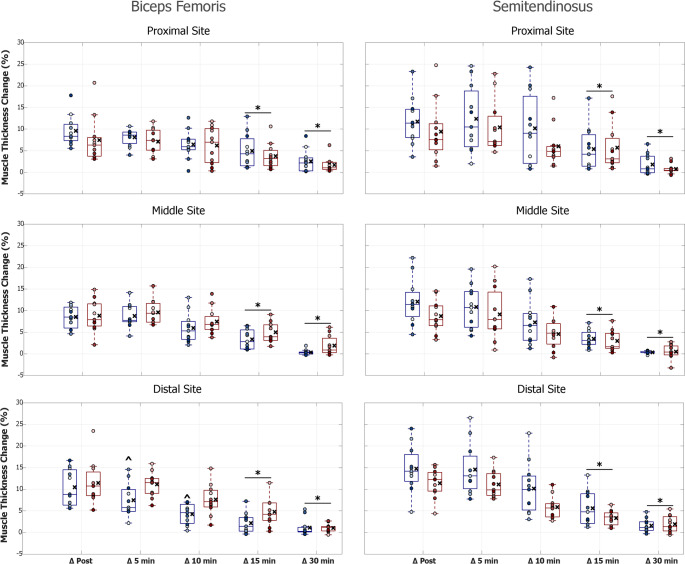
Relative change in muscle thickness (%) from the pre-exercise (*Δ* = [(timepoint − Pre) / Pre] × 100) for biceps femoris (left column) and semitendinosus (right column) at the proximal, middle, and distal measurement sites. Changes are shown immediately post-exercise (*Δ* Post) and at 5-, 10-, 15-, and 30-min post-exercise. Blue and red denote the two sex groups (men and women, respectively). Box boundaries represent the 25_th_ and 75_th_ percentiles (interquartile range), the horizontal line is the median, whiskers extend to values within 1.5 × IQR, circles show individual participants, and × denotes the mean. Individual participant points are color-coded. * significant different than post-exercise (*p* < .05); ^ significant different than ST (*p* < .05);

Analysis indicated significant main effect of *Time* (χ²_(4)_ = 514.99, *p* < .001), *Muscle* (χ²_(1)_ = 17.21, *p* < .001), and *Site* (χ²_(2)_ = 8.83, *p* = .012) for relative change in muscle thickness. Also, significant *Sex* × *Muscle* (χ²_(1)_ = 18.54, *p* < .001), *Muscle × Site* (χ²_(2)_ = 8.16, *p* = .017), *Muscle* × *Time* (χ²_(4)_ = 12.66, *p* = .013) and *Sex* × *Muscle* × *Site* interactions (χ²_(2)_ = 6.98, *p* = .031) were also observed (Fig. 3). Remaining interactions were not significant (*p* ≥ .19). Collapsed across factors, relative change was highest immediately post-exercise (10.4 ± 4.9%, 95% CI [9.5, 11.2]) and declined by 30-min (1.3 ± 1.8%, 95% CI [1.0, 1.6]). Also, the change in muscle thickness of all muscles, sites and sex was lower in 30-min than post-exercise (all *p* ≤ .020, *d* = 1.11–3.74) and 15-min lower than post-exercise (all *p* ≤ .020, *d* = 1.39–2.83). In males, the increase in relative muscle thickness was greater for ST than BF only at the distal site at 5-min (*Δ* = 7.11%, 95% CI [3.8, 10.4], *p* < .001, *d* = 1.01) and 10-min post-exercise (*Δ* = 5.9%, 95% CI [2.6, 9.2], *p* = .013, *d* = 0.79). No other ST–BF or female–male contrasts were significant (all *p* ≥ .05). Detailed descriptive statistics can be found on Supplementary File 3.

### Inter-individual variability in regional contributions

**Fig. 4:**
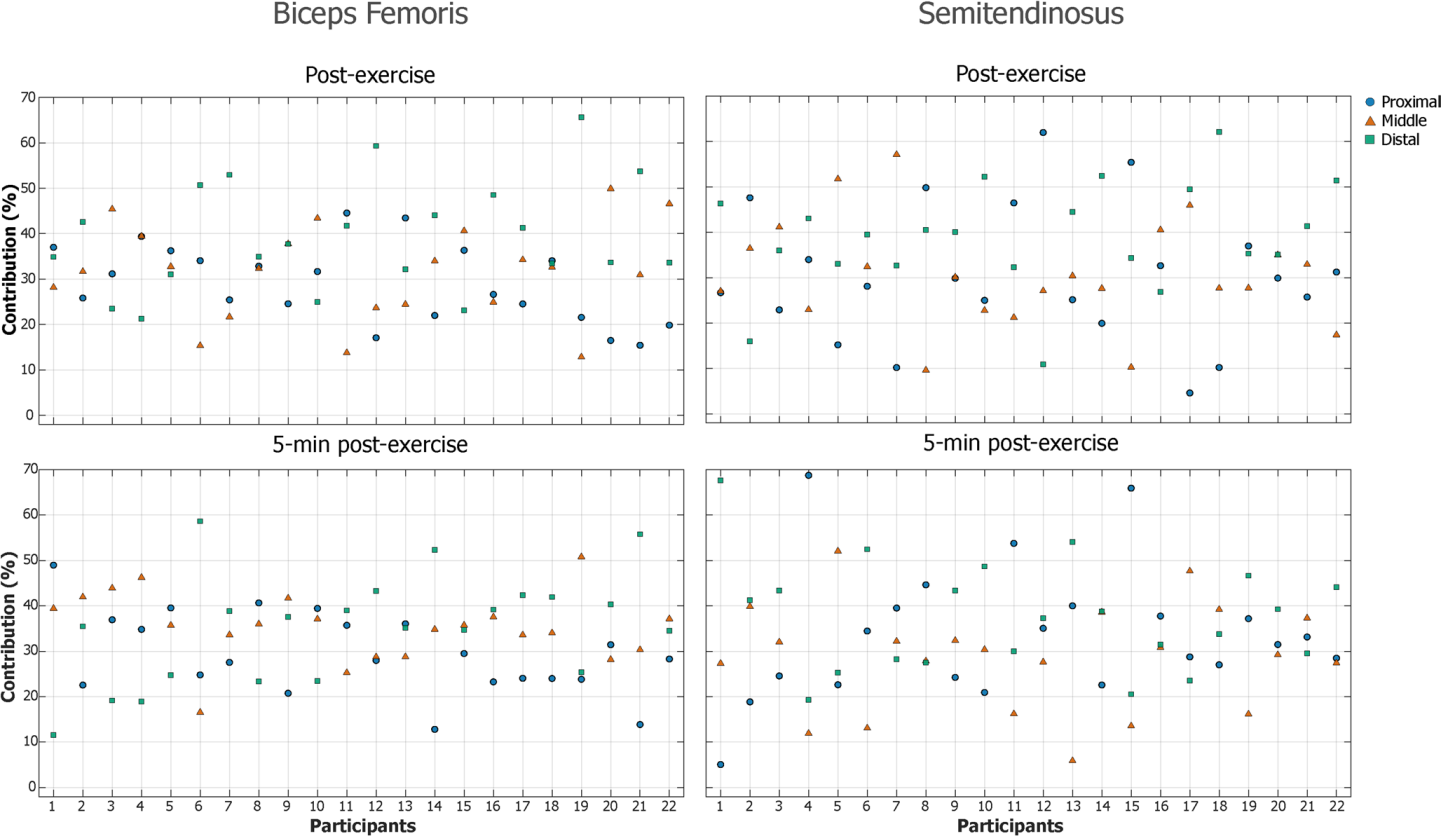
Inter-individual distribution of regional contributions to acute hamstring muscle thickness change. Scatter plots show, for each participant (x-axis), the percentage contribution of the proximal (circles), middle (triangles), and distal (squares) sites to the total change in muscle thickness (y-axis, %). Data are presented for the biceps femoris (left column) and semitendinosus (right column) immediately post-exercise (top row) and 5-min post-exercise (bottom row). For each muscle and time point, site-specific contributions were calculated as |ΔThickness_site| / Σ|ΔThickness| × 100, such that the three sites sum to 100% within each participant

Typical deviations (median and IQR) immediately post-exercise were 7.11 pp [4.04–10.13] (proximal), 7.78 pp [1.60–12.45] (middle), and 7.01 pp [3.19–14.06] (distal). The overall deviation index was 13.15 pp [6.56–16.24] immediately post-exercise and 9.19 pp [6.13–15.84] at 5-min post-exercise. For ST, typical deviations immediately post-exercise were 7.04 pp [3.35–18.73] (proximal), 6.10 pp [1.84–12.06] (middle), and 6.62 pp [3.95–12.25] (distal) and the overall deviation index was 12.10 pp [6.07–21.54] immediately post-exercise and 11.85 pp [8.09–20.65] at 5-min post-exercise. Contribution classification (largest regional contribution per participant) showed that, for BF, the distal region was most frequently contributed to the total change at both timepoints (10/22, 45.5%), followed by middle (7/22, 31.8%) and proximal (5/22, 22.7%) (Fig. 4). For ST, distal region contribution was also most common at both timepoints (12/22, 54.5%), followed by proximal (6/22, 27.3%) and middle (4/22, 18.2%).

### Time-to-peak of acute swelling

**Fig. 5:**
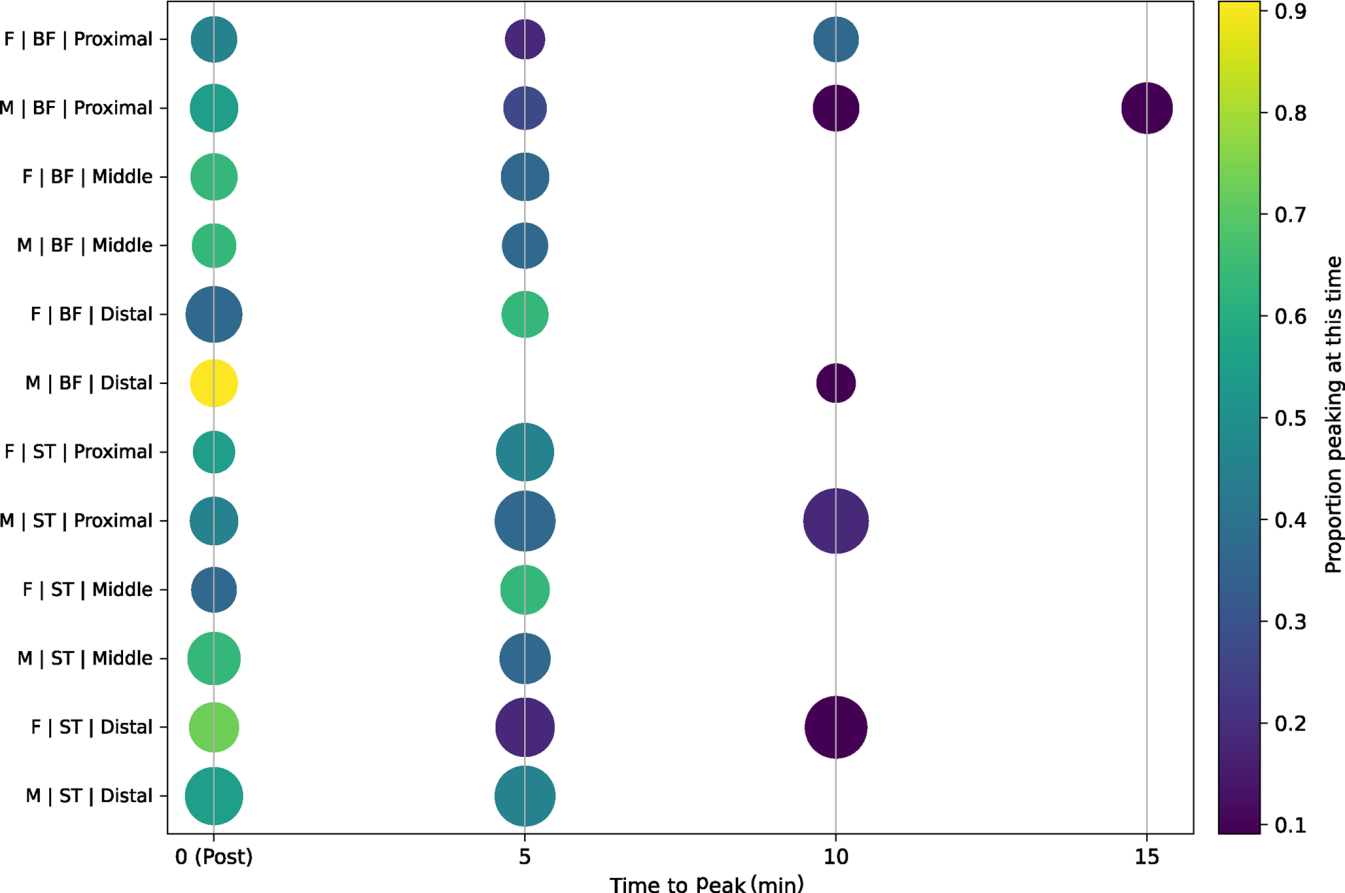
Time to peak of muscle thickness change distribution across sex, muscle, and measurement site. Each row represents one condition and each column a time-to-peak category (post-exercise, 5-, 10-, and 15-min). Bubble color indicates the proportion of participants whose peak occurred at that time point (see color bar). Larger and warmer-colored bubbles therefore indicate a more frequent peak timing and a greater mean peak magnitude within that timing category

Peak *Δ*Thickness occurred most frequently immediately post-exercise (56.8%, 75/132), followed by 5-min (35.6%, 47/132) (Fig. 5). Peaks were uncommon at 10-min (6.8%, 9/132) and 15-min (0.8%, 1/132), and no curves peaked at 30-min (0/132). Multinomial model did not indicate differences in the probability of peaking at post-exercise, 5-min, or ≥ 10-min as a function of *Sex* (χ²_(8)_ = 4.42, *p*=.82), *Muscle* (χ²_(8)_ = 5.94, *p*=.65), and *Site* (χ²_(12)_ = 15.80, *p*=.20) or their interactions (*Sex* × *Muscle*, χ²_(2)_ = 1.83, *p*=.40; *Sex × Site*, χ²_(4)_ = 0.78, *p*=.94; *Muscle × Site*, χ²_(4)_ = 0.99, *p*=.91). Model-estimated predicted probabilities were consistent with the post-exercise ranging from 0.44 to 0.78 and 5-min ranging from 0.19 to 0.52, while for ≥ 10 min was generally low.

### Correlation between mechanical work and thickness change

No significant correlation was observed between total mechanical work and muscle thickness change post-exercise in BF (proximal: *r* = .36, *p* = .088; middle: *r* = .03, *p* = .73; distal: *r* = .21, *p* = .33) and ST (proximal: *r* = .28, *p* = .19; middle: *r* = .26, *p* = .23; distal: *r* = .09, *p* = .69) (Supplementary File 4).

## Discussion

This study quantified the time course of hamstrings muscle thickness following maximal concentric exercise, focusing on the influence of muscle, measurement site and sex on absolute and relative swelling magnitudes. First, maximal isokinetic exercise induced a transient increase in hamstring thickness that peaked by 5-min and resolved by 30-min post-exercise. Second, we observed significant intra- and inter-muscular differences in absolute thickness increases (BF > ST; middle > proximal/distal), though relative responses remained mostly similar across sites and muscles despite substantial inter-individual variability in the regional contribution to the overall response. Third, sex differences were evident in absolute thickness (males > females) but disappeared upon normalization to baseline thickness.

### Time course of swelling following concentric exercise

The fast rise and the quick return toward baseline of muscle thickness (Figs. 2 and 3) are in line with our initial hypothesis and with reports from other muscle groups showing that muscle size increases are largest immediately after exercise and fade substantially within the first 15–30 min (Csapo et al. 2011; Freitas et al. 2020; Arima et al. 2023). The most likely explanation for this early time course is a transient physiological response rather than swelling from tissue damage. A rapid increase in blood flow to working muscle occurs at the onset of exercise, driven by higher metabolic demand and vascular regulation (Leyk et al. 1994; Ploutz-Snyder et al. 1995; Finan and Guilak 2010). That hyperemia, together with changes in hydrostatic and osmotic forces, can increase muscle volume and therefore measured thickness shortly after exercise (Lanne et al. 1959; Leyk et al. 1994; Finan and Guilak 2010). Inflammation-related edema linked to muscle damage is typically expected to become more visible later, not within minutes. Thus, the early increase in muscle thickness observed in the present study is most consistent with a short-lived exercise-induced fluid shift rather than a marker of muscle damage per se (Leyk et al. 1994; Ploutz-Snyder et al. 1995), although the underlying mechanisms were not directly examined.

Work examining body-fluid compartments supports this view (Sjogaard and Saltin 1982; Sjogaard et al. 1985; Raja et al. 2006). For example, Raja et al. (2006) reported rapid shifts during and immediately after resistance exercise, including quick normalization of intracellular water and a tendency for extracellular water to rise during early recovery. Earlier biopsy and tracer work also suggests that total water and extracellular water within muscle tissue can increase after intense exercise, with smaller or less consistent changes in intracellular water (Sjogaard and Saltin 1982; Sjogaard et al. 1985).

### Intra- and inter-muscular responses following concentric exercise

Partly, consistent with our secondary hypothesis, significant, muscle- and region-specific changes in absolute thickness were observed after exercise (Fig. 2). Overall, the BF showed larger increases than ST and these changes were greater at middle site, followed by proximal, then distal. In contrast, when expressed as relative change, the acute response was broadly similar across muscles and sites (Fig. 3). This distinction likely reflects that absolute changes are influenced by baseline muscle thickness, whereas relative changes represent the proportional response normalized to initial size. The only exception was the distal ST in men, which increased more than distal BF at 5 and 10 min (~ 7% and ~ 6% greater, respectively). This pattern is consistent with previously reported intra- and inter-muscular heterogeneity in muscle architecture (Kellis 2018; Sahinis et al. 2025c) and tendon mechanical properties in hamstrings (Kellis and Sahinis 2022; Sahinis and Kellis 2024b). These architectural and mechanical differences may alter how force and strain are distributed along the muscle–tendon unit, potentially creating region-specific intramuscular pressure and perfusion patterns that influence local fluid accumulation and the resulting thickness response. In addition, BF and ST (Sahinis et al. 2025b) and the proximal and distal regions of ST (Sahinis et al. 2024) receive partly independent neural input, which may enable muscle- and region-specific control and contribute to distinct acute responses. The lack of differences in normalized values likely reflects that thicker regions at rest show larger absolute increases despite similar proportional responses, so normalization minimizes baseline size effects and reducing between-region differences.

One interesting finding is that all the participants did not show the same regional pattern of change. Even if average responses look similar across regions (Fig. 3), some individuals may show a more proximal or more distal site change (see for example participant 8 and 6 in Fig. 4). Although the distal region most frequently accounted for the largest share of the total response, a substantial proportion of participants showed a greater proximal or middle contribution, indicating that a single “typical” regional pattern does not apply to everyone. Consistent with this, the distribution-based variability metrics show participant-specific deviations from the group median shares (by several percentage points across sites), and the total variation distance further indicates heterogeneity in swelling distributions across individuals. If these individual profiles are repeatable across sessions, they may help explain why some longitudinal studies report region-specific adaptations despite modest regional differences at the group level. Collectively, these observations support multi-site assessment when characterizing hamstring morphology and acute responses, as single-site measures may not capture the spatial distribution of thickness change.

### Sex differences following concentric exercise

Absolute thickness responses were smaller in women than in men (Fig. 2), but this sex difference disappeared after normalization to baseline thickness, in line with our third hypothesis (Fig. 3). This indicates that the sex effect was present for the absolute magnitude of thickening, but not for the proportional response once baseline size was taken into account. Together, these findings suggest that the early hemodynamic/fluid response to a standardized maximal task may be similar between sexes despite differences in muscle size. These results are in line with the Freitas et al. (2020) that reported greater change in absolute muscle thickness in men than women after blood flow restriction training. The absence of differences in relative outcomes is consistent with evidence that men and women exhibit comparable relative training-induced hypertrophy (Abe et al. 2000; Roberts et al. 2020; Refalo et al. 2025), although the present acute swelling response should not be interpreted as a direct indicator of long-term structural adaptation.

### Limitations

Several limitations should be considered when interpreting these findings. Acute thickness responses were assessed during a controlled, maximal concentric fatiguing isokinetic task; therefore, the findings may differ with other contraction modes, velocities, or sport-specific movements. However, the use of isokinetic dynamometry strengthens the study by fixing movement speed and range of motion and providing a direct, reproducible measure of torque/effort, making it well suited for isolating time-course and site-specific effects. A second limitation is that the protocol was knee-dominant, which may preferentially stress regions most involved in knee torque and therefore may not generalize to multi-joint or hip-dominant tasks (e.g., stiff-leg deadlift, sprinting, or combined hip–knee actions) (Sahinis et al. 2025a). Third, participants were physically active young adults rather than trained athletes, and so the magnitude and time course of swelling in athletic populations remain unclear. Thickness was quantified from longitudinal two-dimensional ultrasound images, which do not capture the tissue’s three-dimensional morphology; accordingly, these estimates may be sensitive to the scanning path and probe alignment. An additional limitation is that the protocol was based on a fixed number of maximal contractions rather than being work-matched, resulting in greater total mechanical work in males than females. Nevertheless, total work was not significantly correlated with changes in muscle thickness (Supplementary File 4), indicating that variation in workload across participants is unlikely to explain the swelling responses observed here. Finally, although between-participant variability in regional responses was observed, the reproducibility of these individual patterns across sessions was not assessed, nor was their association with underlying neural activation or muscle architecture.

### Practical implications

Our findings provide some practical points for coaches and trainers. First, since muscle swelling peaks at 5 min and mostly disappears by 30 min, practitioners aiming to maximize metabolic stress should keep rest periods short and transitions between exercises quick. Second, BF showed a greater absolute increase in muscle thickness than ST, and the middle site showed a greater absolute increase than the proximal and distal sites. Interestingly, the regional pattern responses varied significantly between individuals, suggesting that single-site measurements may not capture the spatial responses. Third, men showed larger absolute increases in muscle thickness, but the relative (% change) response was similar between sexes. This suggests that training does not need to be adjusted by sex when the focus is the acute responses. These points are based on acute measurements after maximal concentric contractions performed on an isokinetic dynamometer. Future studies should test whether the same time course and regional differences occur with common field-based hamstring exercises and whether they relate to longer-term training adaptations.

## Conclusion

Maximal concentric isokinetic exercise produced a rapid increase in hamstring muscle thickness that was largest withing 5-min post-exercise, and then returned close to baseline by 15–30 min. Absolute thickness changes showed intra- and inter-muscular differences, whereas relative (percentage) changes were largely comparable between sexes and across measurement sites, yet exhibited considerable inter-individual variability in the distribution of swelling along the muscle length. Acute hamstring swelling is highly time dependent and region specific. Protocols should standardize post-exercise measurement timing, and analyses should characterize individual regional response patterns rather than relying solely on group means.

## Supplementary Information

Below is the link to the electronic supplementary material.

Supplementary Material 1

Supplementary Material 2

Supplementary Material 3

Supplementary Material 4

## Data Availability

The data supporting the findings of this study are available upon reasonable request from the corresponding author.

## Abbreviations

ANOVA: Analysis of variance
BF: Biceps femoris
CI: Confidence interval
LMM: Linear mixed-effects model
ST: Semitendinosus
