# Supplementary material for: Time course and regional heterogeneity of hamstring muscle thickness after maximal concentric exercise in men and women
Source: Eur J Appl Physiol. 2026 Apr 13;126(7):3929–40. doi: 10.1007/s00421-026-06223-8 (PMC13380580; doi:10.1007/s00421-026-06223-8)
Supplement: Supplementary file 1 — Supplementary Material 1 [file 421_2026_6223_MOESM1_ESM.docx]

**Supplementary File 1.**

**Methods**

***Reliability procedures***

Reliability of the ultrasound thickness measurements was assessed in a subset of 10 (5 men and 5 women) participants (mean ± SD, men, age 22.1 ± 1.4 y, mass 82.1 ± 6.1 kg, height 1.80 ± 0.06 m; women, age 22.0 ± 1.1 y, mass 71.0 ± 4.3 kg, height 1.68 ± 0.07 m). Measurements were obtained by two investigators (C.S. and K.P.) using two ultrasound systems (SSD-3500, ALOKA, Japan; and GE LOGIQ 400 CL PRO, GE Medical Systems, U.K.) equipped with a 10-MHz linear-array transducer (6-cm footprint). For intra-rater reliability, the same examiner (C.S.) repeated the full measurement procedure after a 30-min interval during the same session, including probe repositioning and reacquisition of the images. For inter-rater reliability, the second examiner (K.P.) independently repeated the same measurement procedure under identical conditions. For inter-device reliability, the same anatomical sites were assessed using both ultrasound systems during the same session under the same experimental setup. Reliability was evaluated for the proximal (40%), middle (60%), and distal (80%) sites of the semitendinosus (ST) and biceps femoris long head (BF).

*Statistical analysis*

Reliability was assessed using intraclass correlation coefficients (ICC) based on two-way models with absolute agreement. Intra-rater and inter-device reliability were evaluated using a two-way mixed-effects model (ICC₃.₁), whereas inter-rater reliability was assessed using a two-way random-effects model (ICC₂.₁). Absolute reliability was quantified using the standard error of measurement (SEM), calculated as: **SEM = SD × √(1 − ICC)** where *SD* represents the pooled standard deviation of the measurements. Agreement between measurements was further examined using Bland–Altman analysis, with bias defined as the mean difference between measurements and 95% limits of agreement (LoA) calculated as bias ± 1.96 × SD of the differences. Reliability coefficients were interpreted according to established criteria: < .50 poor, .50–.75 moderate, .75–.90 good, and > .90 excellent reliability.

**Results**

*Intra-rater reliability*

Test–retest reliability was high across all regions for both muscles (ICC₃.₁ = 0.90–0.97), with low absolute measurement error (SEM = 0.54–0.75 mm). Systematic bias between sessions was minimal (−0.08 to 0.44 mm). Bland–Altman limits of agreement were narrow across sites, ranging from −2.26 to 2.31 mm (Table 1).

**Table 1:** Intra-rater reliability values (*n* = 10).

|  | Test (mm) | Re-test (mm) | | ICC_3.1_ | SEM | Bias ± L-LoA | U-LoA |  |
| --- | --- | --- | --- | --- | --- | --- | --- | --- |
| Proximal Region | |  |  | |  |  |  | |
| ST | 28.5 ± 3.5 | 28.4 ± 3.1 | 0.97 | | 0.59 | -0.08 ± -1.85 | 1.69 | |
| BF | 31.6 ± 2.7 | 32.0 ± 3.0 | 0.94 | | 0.69 | 0.44 ± -1.42 | 2.31 | |
| Middle region | |  |  | |  |  |  | |
| ST | 32.0 ± 3.0 | 32.1 ± 3.5 | 0.97 | | 0.54 | 0.13 ± -1.46 | 1.72 | |
| BF | 38.1 ± 2.9 | 38.2 ± 2.9 | 0.93 | | 0.72 | 0.14 ± -2.00 | 2.28 | |
| Distal region | |  |  | |  |  |  | |
| ST | 21.4 ± 2.6 | 21.7 ± 3.0 | 0.96 | | 0.54 | 0.34 ± -1.10 | 1.79 | |
| BF | 25.1 ± 2.3 | 25.1 ± 2.5 | 0.90 | | 0.75 | -0.03 ± -2.26 | 2.2 | |

ST: Semitendinosus; BF: Biceps Femoris

*Inter-rater reliability*

Inter-rater reliability was good to excellent across all regions and muscles. ICC values ranged from 0.87 to 0.95, with SEM values between 0.61 and 1.00 mm. Systematic bias between raters was small (−0.39 to 0.63 mm). Bland–Altman limits of agreement ranged from −3.16 to 3.24 mm (Table 2).

**Table 2:** Inter-rater reliability values (*n* = 10).

|  | Test (mm) | Re-test (mm) | | ICC_2.1_ | SEM | Bias ± L-LoA | U-LoA |  |
| --- | --- | --- | --- | --- | --- | --- | --- | --- |
| Proximal Region | |  |  | |  |  |  | |
| ST | 28.5 ± 3.5 | 28.8 ± 3.8 | 0.93 | | 0.96 | 0.34 ± -2.45 | 3.13 | |
| BF | 31.6 ± 2.7 | 31.9 ± 3.3 | 0.94 | | 0.69 | 0.29 ± -1.70 | 2.27 | |
| Middle region | |  |  | |  |  |  | |
| ST | 32.0 ± 3.0 | 32.3 ± 3.4 | 0.9 | | 1.00 | 0.35 ± -2.55 | 3.24 | |
| BF | 38.1 ± 2.9 | 37.7 ± 2.5 | 0.87 | | 0.97 | -0.39 ± -3.16 | 2.39 | |
| Distal region | |  |  | |  |  |  | |
| ST | 21.4 ± 2.6 | 21.4 ± 3.2 | 0.95 | | 0.61 | 0.00 ± -1.83 | 1.83 | |
| BF | 25.1 ± 2.3 | 25.7 ± 2.2 | 0.91 | | 0.67 | 0.63 ± -0.90 | 2.17 | |

ST: Semitendinosus; BF: Biceps Femoris

*Inter-device reliability*

Inter-device reliability was excellent across all regions and muscles. ICC values ranged from 0.94 to 0.99, with low measurement error (SEM = 0.23–0.67 mm). Systematic bias between devices was minimal (−0.06 to 0.43 mm). Bland–Altman limits of agreement ranged from −2.07 to 1.94 mm (Table 3).

**Table 3:** Inter-device reliability values (*n* = 10).

|  | Test (mm) | Re-test (mm) | | ICC_3.1_ | SEM | Bias ± L-LoA | U-LoA |  |
| --- | --- | --- | --- | --- | --- | --- | --- | --- |
| Proximal Region | |  |  | |  |  |  | |
| ST | 28.5 ± 3.5 | 28.4 ± 3.9 | 0.96 | | 0.67 | -0.06 ± -2.07 | 1.94 | |
| BF | 31.6 ± 2.7 | 31.8 ± 3.1 | 0.97 | | 0.49 | 0.19 ± -1.25 | 1.62 | |
| Middle region | |  |  | |  |  |  | |
| ST | 32.0 ± 3.0 | 32.4 ± 2.9 | 0.97 | | 0.47 | 0.43 ± -0.68 | 1.53 | |
| BF | 38.1 ± 2.9 | 38.4 ± 3.0 | 0.96 | | 0.58 | 0.29 ± -1.33 | 1.92 | |
| Distal region | |  |  | |  |  |  | |
| ST | 21.4 ± 2.6 | 21.4 ± 2.5 | 0.99 | | 0.23 | -0.05 ± -0.75 | 0.64 | |
| BF | 25.1 ± 2.3 | 25.1 ± 2.8 | 0.94 | | 0.59 | -0.03 ± -1.80 | 1.74 | |

ST: Semitendinosus; BF: Biceps Femoris
