# Supplementary material for: Time course and regional heterogeneity of hamstring muscle thickness after maximal concentric exercise in men and women
Source: Eur J Appl Physiol. 2026 Apr 13;126(7):3929–40. doi: 10.1007/s00421-026-06223-8 (PMC13380580; doi:10.1007/s00421-026-06223-8)
Supplement: Supplementary file 2 — Supplementary Material 2 [file 421_2026_6223_MOESM2_ESM.docx]

**Supplementary File 2.**

Mean (SD) and 95% confidence intervals (CI) for muscle thickness (mm) stratified by sex (male, female), muscle (biceps femoris long head, BF; semitendinosus, ST), and site (proximal, middle, distal) across time points (Pre, Post, 5, 10, 15, and 30 min).

| **Muscle** | **Sex** | **Time points** | | | | | |
| --- | --- | --- | --- | --- | --- | --- | --- |
|  |  | **Pre-exercise** | **Post-exercise** | **5-min** | **10-min** | **15-min** | **30-min** |
|  |  | **Proximal site** | | | | | |
| **BF** | **Males** | 32.3 ± 3.5 (29.9, 34.7) | 35.4 ± 3.8 (32.8, 37.9) | 34.9 ± 3.6 (32.5, 37.3) | 34.4 ± 3.8 (31.8, 37.0) | 33.9 ± 3.7 (31.4, 36.4) | 33.1 ± 3.5 (30.7, 35.4) |
|  | **Females** | 29.9 ± 1.9 (28.6, 31.1) | 32.1 ± 2.8 (30.2, 34.0) | 32.0 ± 2.5 (30.4, 33.7) | 31.8 ± 2.6 (30.0, 33.5) | 31.0 ± 2.2 (29.5, 32.5) | 30.4 ± 1.6 (29.3, 31.5) |
| **ST** | **Males** | 29.3 ± 3.8 (26.7, 31.9) | 32.7 ± 4.2 (29.8, 35.5) | 33.1 ± 4.8 (29.9, 36.3) | 32.2 ± 4.2 (29.4, 35.0) | 30.9 ± 4.2 (28.1, 33.8) | 29.8 ± 3.8 (27.3, 32.3) |
|  | **Females** | 25.4 ± 2.9 (23.5, 27.3) | 27.7 ± 2.6 (25.9, 29.5) | 28.3 ± 3.3 (26.0, 30.5) | 27.0 ± 2.8 (25.1, 28.9) | 26.9 ± 3.1 (24.8, 29.0) | 25.6 ± 2.8 (23.7, 27.5) |
|  |  | **Middle site** | | | | | |
| **BF** | **Males** | 38.3 ± 3.6 (35.9, 40.7) | 41.6 ± 4.2 (38.7, 44.4) | 41.6 ± 4.0 (38.9, 44.3) | 40.6 ± 4.1 (37.8, 43.3) | 39.6 ± 4.1 (36.8, 42.3) | 38.4 ± 3.6 (36.0, 40.8) |
|  | **Females** | 36.3 ± 2.8 (34.4, 38.2) | 39.5 ± 3.2 (37.3, 41.6) | 39.8 ± 3.1 (37.7, 41.9) | 39.0 ± 3.1 (36.9, 41.1) | 38.3 ± 3.4 (36.0, 40.6) | 37.0 ± 3.0 (35.0, 39.0) |
| **ST** | **Males** | 32.0 ± 3.8 (29.5, 34.6) | 35.9 ± 4.3 (33.0, 38.8) | 35.4 ± 3.4 (33.1, 37.6) | 34.3 ± 4.1 (31.6, 37.1) | 33.3 ± 3.9 (30.7, 35.9) | 32.1 ± 3.8 (29.6, 34.7) |
|  | **Females** | 27.4 ± 2.8 (25.5, 29.2) | 29.7 ± 2.8 (27.8, 31.6) | 29.8 ± 3.4 (27.6, 32.1) | 28.6 ± 2.7 (26.7, 30.4) | 28.1 ± 2.5 (26.5, 29.8) | 27.5 ± 2.8 (25.6, 29.4) |
|  |  | **Distal site** | | | | | |
| **BF** | **Males** | 25.9 ± 4.4 (23.0, 28.9) | 28.6 ± 4.9 (25.3, 31.9) | 27.8 ± 4.7 (24.7, 31.0) | 27.0 ± 4.6 (23.9, 30.1) | 26.5 ± 4.4 (23.5, 29.4) | 26.1 ± 4.4 (23.2, 29.1) |
|  | **Females** | 23.5 ± 4.1 (20.7, 26.2) | 26.0 ± 4.1 (23.3, 28.8) | 26.0 ± 4.3 (23.1, 28.9) | 25.3 ± 4.4 (22.3, 28.2) | 24.6 ± 4.2 (21.8, 27.4) | 23.7 ± 4.1 (20.9, 26.4) |
| **ST** | **Males** | 21.5 ± 3.2 (19.4, 23.7) | 24.6 ± 3.2 (22.5, 26.8) | 24.6 ± 3.6 (22.2, 27.1) | 23.7 ± 3.3 (21.4, 25.9) | 22.7 ± 3.0 (20.7, 24.7) | 21.9 ± 3.3 (19.7, 24.1) |
|  | **Females** | 20.1 ± 2.9 (18.2, 22.0) | 22.4 ± 3.2 (20.2, 24.5) | 22.2 ± 3.0 (20.1, 24.2) | 21.5 ± 2.9 (19.5, 23.5) | 20.8 ± 2.9 (18.8, 22.7) | 20.5 ± 2.9 (18.5, 22.5) |

BF: Biceps femoris; ST: Semitendinosus
