# Supplementary material for: Time course and regional heterogeneity of hamstring muscle thickness after maximal concentric exercise in men and women
Source: Eur J Appl Physiol. 2026 Apr 13;126(7):3929–40. doi: 10.1007/s00421-026-06223-8 (PMC13380580; doi:10.1007/s00421-026-06223-8)
Supplement: Supplementary file 3 — Supplementary Material 3 [file 421_2026_6223_MOESM3_ESM.docx]

**Supplementary File 3.**

Mean (SD) and 95% confidence intervals (CI) for relative change in muscle thickness (%) stratified by sex (male, female), muscle (biceps femoris long head, BF; semitendinosus, ST), and site (proximal, middle, distal) across time points (Post-exercise, 5, 10, 15, and 30 min).

| **Muscle** | **Sex** | **Time points** | | | | |
| --- | --- | --- | --- | --- | --- | --- |
|  |  | **Post-exercise** | **5-min** | **10-min** | **15-min** | **30-min** |
|  |  | **Proximal site** | | | | |
| **BF** | **Males** | 9.6±3.5 (7.2, 11.9) | 8.1±2.0 (6.8, 9.4) | 6.4±3.3 (4.2, 8.6) | 4.9±4.0 (2.3,7.6) | 2.5±2.6 (0.8, 4.3) |
|  | **Females** | 7.4±5.3 (3.9, 11.0) | 7.1±2.9 (5.1, 9.0) | 6.2±4.2 (3.4, 9.0) | 3.7±2.9 (1.8, 5.7) | 1.7±1.7 (0.6, 2.9) |
| **ST** | **Males** | 11.7±5.5 (8.1, 15.4) | 13.0±9.0 (7.0, 19.0) | 10.2±8.2 (4.7, 15.7) | 5.4±5.1 (1.9, 8.8) | 1.8±2.4 (0.2, 3.4) |
|  | **Females** | 9.4±6.8 (4.9, 14.0) | 11.3±8.1 (5.9, 16.8) | 6.5±5.9 (2.5, 10.4) | 5.7±5.5 (2.0, 9.4) | 0.8±1.1 (0.1, 1.5) |
|  |  | **Middle site** | | | | |
| **BF** | **Males** | 8.5±2.5 (6.8, 10.2) | 8.8±2.8 (6.9, 10.6) | 6.0±3.4 (3.7, 8.3) | 3.4±2.2 (1.9, 4.9) | 0.3±0.6 (-0.1, 0.7) |
|  | **Females** | 8.8±3.7 (6.3, 11.3) | 9.6±2.8 (7.7, 11.5) | 7.5±3.0 (5.4, 9.5) | 5.0±2.3 (3.4, 6.5) | 1.9±2.2 (0.4, 3.4) |
| **ST** | **Males** | 12.1±5.3 (8.5, 15.6) | 10.8±4.9 (7.5, 14.1) | 7.3±5.1 (3.8, 10.8) | 3.5±2.0 (2.2, 4.8) | 0.4±0.3 (0.2, 0.6) |
|  | **Females** | 8.7±3.7 (6.3, 11.2) | 9.1±6.1 (5.1, 13.2) | 4.6±3.4 (2.3, 6.9) | 3.0±2.4 (1.4, 4.6) | 0.5±1.6 (-0.6, 1.6) |
|  |  | **Distal site** | | | | |
| **BF** | **Males** | 10.5±4.2 (7.6, 13.3) | 7.5±3.8 (4.9, 10.0) | 4.2±2.3 (2.7, 5.8) | 2.2±2.3 (0.6, 3.7) | 0.9±2.3 (-0.6-2.4) |
|  | **Females** | 11.5±4.9 (8.1, 14.8) | 11.1±2.8 (9.2, 13.0) | 7.6±3.6 (5.2, 10.0) | 4.8±3.2 (2.7, 6.9) | 0.8±0.8 (0.3-1.4) |
| **ST** | **Males** | 14.7±5.4 (11.1, 18.3) | 14.6±5.9 (10.6, 18.5) | 10.2±6.0 (6.1, 14.2) | 5.6±4.0 (2.9, 8.3) | 1.6±1.5 (0.6-2.6) |
|  | **Females** | 11.4±3.4 (9.1, 13.6) | 10.5±5.7 (6.7, 14.4) | 7.1±5.0 (3.8, 10.5) | 3.3±1.7 (2.2, 4.5) | 1.9±1.9 (0.6-3.2) |

BF: Biceps Femoris; ST: Semitendinosus
