# Supplementary material for: Time course and regional heterogeneity of hamstring muscle thickness after maximal concentric exercise in men and women
Source: Eur J Appl Physiol. 2026 Apr 13;126(7):3929–40. doi: 10.1007/s00421-026-06223-8 (PMC13380580; doi:10.1007/s00421-026-06223-8)

**Supplementary File 4.**

**Association between mechanical work and post-exercise changes in hamstring thickness collapsed across sexes (n = 22).**

Scatterplots showing the relationship between total mechanical work performed during the concentric fatiguing protocol and the relative change in muscle thickness (%) for the biceps femoris (top row) and semitendinosus (bottom row) at the proximal, middle, and distal measurement sites. Each data point represents an individual participant. The solid line represents the linear regression fit. Pearson correlation coefficients (r) and associated p-values are shown within each panel. No significant associations were observed between total work and thickness changes across muscles or sites.


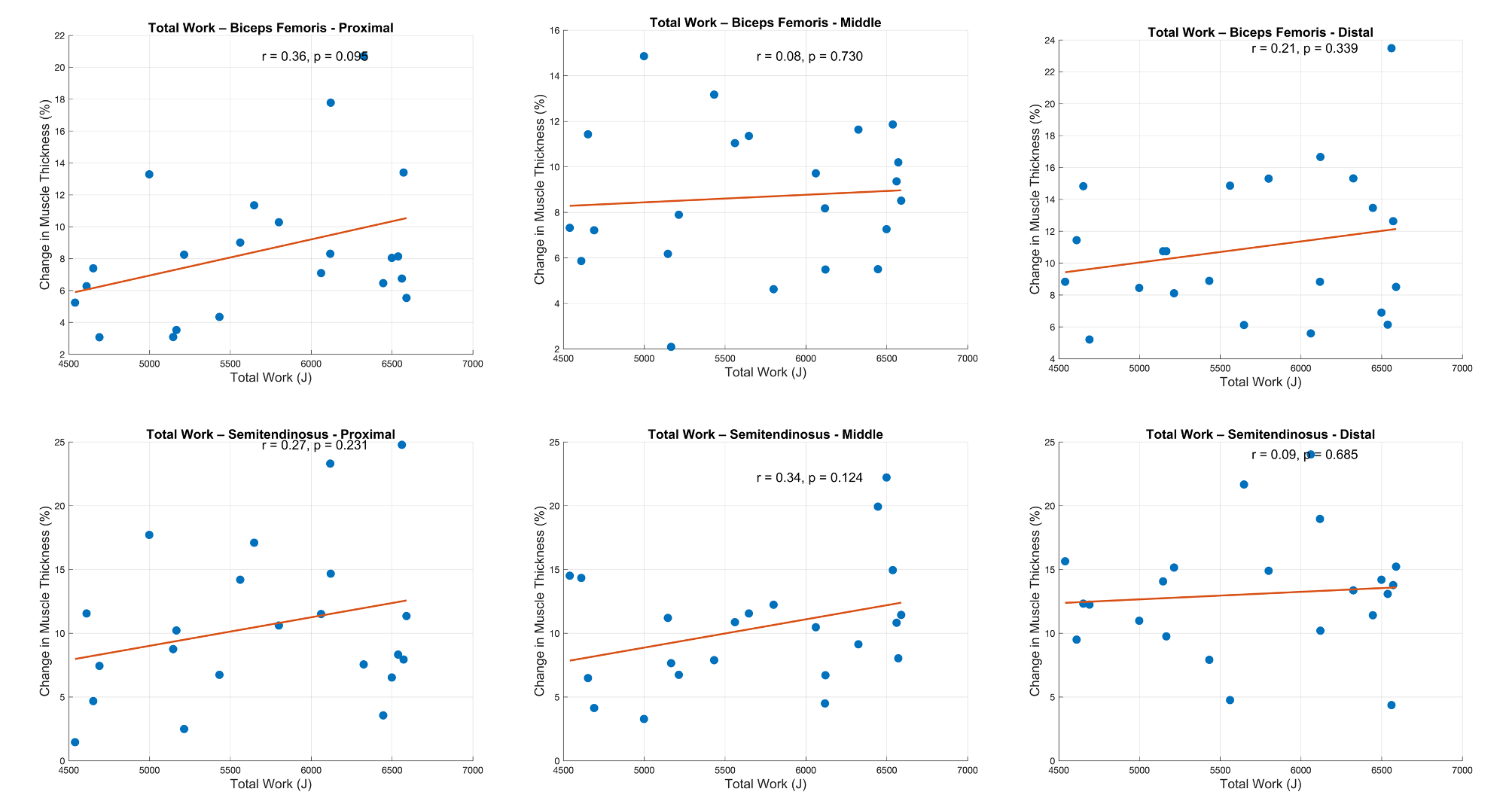

Supplement: Supplementary file 4 — Supplementary Material 4 [file 421_2026_6223_MOESM4_ESM.docx]
